# Recombinant λ-phage nanobioparticles for tumor therapy in mice models

**DOI:** 10.1186/1479-0556-8-3

**Published:** 2010-05-12

**Authors:** Amir Ghaemi, Hoorieh Soleimanjahi, Pooria Gill, Zuhair Hassan, Soodeh Razeghi M Jahromi, Farzin Roohvand

**Affiliations:** 1 Department of Virology, Faculty of Medical Sciences, Tarbiat Modares University, Tehran, 14115-111 Iran; 2 Department of Nanobiotechnology, Faculty of Biological Sciences, Tarbiat Modares University, Tehran, 14115-175 Iran; 3 Department of Immunology, Faculty of Medical Sciences, Tarbiat Modares University, Tehran, 14115-111 Iran; 4 Shefa Neuroscience Research Centre, Tehran, Iran; 5 Hepatitis and AIDS Department, Pasteur Institute, Tehran, Iran; 6 Faculty of Medicine, Golestan University of Medical Sciences and Health Care, Gorgan, Iran

## Abstract

Lambda phages have considerable potential as gene delivery vehicles due to their genetic tractability, low cost, safety and physical characteristics in comparison to other nanocarriers and gene porters. Little is known concerning lambda phage-mediated gene transfer and expression in mammalian hosts. We therefore performed experiments to evaluate lambda-ZAP bacteriophage-mediated gene transfer and expression *in vitro*. For this purpose, we constructed recombinant λ-phage nanobioparticles containing a mammalian expression cassette encoding enhanced green fluorescent protein (EGFP) and E7 gene of human papillomavirus type 16 (λ-HPV-16 E7) using Lambda ZAP- CMV XR vector. Four cell lines (COS-7, CHO, TC-1 and HEK-239) were transduced with the nanobioparticles. We also characterized the therapeutic anti-tumor effects of the recombinant λ-HPV-16 E7 phage in C57BL/6 tumor mice model as a cancer vaccine. Obtained results showed that delivery and expression of these genes in fibroblastic cells (COS-7 and CHO) are more efficient than epithelial cells (TC-1 and HEK-239) using these nanobioparticles. Despite the same phage M.O.I entry, the internalizing titers of COS-7 and CHO cells were more than TC-1 and HEK-293 cells, respectively. Mice vaccinated with λ-HPV-16 E7 are able to generate potent therapeutic antitumor effects against challenge with E7- expressing tumor cell line, TC-1 compared to group treated with the wild phage. The results demonstrated that the recombinant λ-phages, due to their capabilities in transducing mammalian cells, can also be considered in design and construction of novel and safe phage-based nanomedicines.

## Introduction

Different strategies have been employed for gene delivery and expression in mammalian cells. Two main types of these strategies are viral and non-viral vectors [1,2]. The most known viral vehicles having been effectively employed as gene transfer vectors in vitro include the vaccinia viruses [3], herpes simplex viruses[4], adenoviruses[5], influenza viruses [6], lentiviruses [7] retroviruses [8], and adeno-associated viruses. Non-viral vehicles include polymers (condensing and non-condensing ones)[9], bacterial spores[10], proteosomes [11], exosomes [12], liposomes [13], virosomes [14], superfluids [15], nanoparticle-based nanobeads [16], virus-liked particles [17] and bacteriophages [18].

The application of bacteriophages for gene delivery and vaccination has already been described [19,20]. Particularly, lambda bacteriophages having various appealing characteristics as gene/vaccine delivery vehicles, possess a high degree of stability [21], high production capacity [22], compatibility with rapid and inexpensive production or purification methods [23], genetic tractability and inherent biological safety in mammalian cells [24].

The dimensions of the lambda phage particles are broadly similar to those of many mammalian viruses and recent structural evidence points to a shared ancestry between tailed bacteriophages and mammalian DNA viruses [25]. However, the eukaryotic cell poses numerous barriers to phage-mediated gene transfer[25]. After macropinocytosis and internalization, phage must gain access to the cytoplasm, uncoat and deliver its DNA payload to the nuclei.

Since little is known concerning phage-mediated gene transfer in mammalian cells [26], we therefore performed experiments to examine phage-mediated gene transfer *in vitro*. For this purpose, recombinant lambda phages were characterized to compare their performance for gene delivery and expression in four cell lines from different sources.

The discovery that human papillomavirus (HPV) causes the vast majority of cervical cancers opens exciting new possibilities for controlling this disease, which is the second most common cancer among women worldwide. Vaccines that protect against HPV infection, if administered prior to initiation of sexual activity, theoretically would prevent women from developing cervical cancer later in life.

Since human papillomavirus (HPV) causes the vast majority of cervical cancers, exciting new approaches for controlling the disease were performed. Therapeutic vaccines are aimed at promoting regression of HPV-associated lesions by the induction of cellular immune responses directed against viral proteins expressed in tumor cells[25].

Human HPV-16 E7 was also chosen for vaccine development because HPVs, particularly HPV-16, are associated with most cervical cancers. The HPV oncogenic proteins, E6 and E7, are important in the induction and maintenance of cellular transformation and coexpressed in most HPV-containing cervical cancers. Vaccines or immunological therapeutics targeting E7 and/or E6 proteins may provide an opportunity to treat HPV-associated cervical malignancy [25]. In the present study, we have accordingly taken advantage of the bacteriophage Lambda as a gene delivery vector for HPV-16 E7 and evaluate anti-tumor effects of the phage in C57BL/6 mice. The results potentially confirmed the capability of these biological tools in offering new therapeutic strategies against TC-1 tumors in mice.

## Materials and methods

### Lambda vector

**Figure 1:**
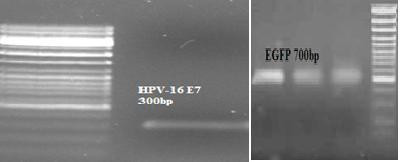
**A) Presence of EGFP gene (700 bp) in packaged phages was confirmed using PCR**. Lane 1 is negative control. Lane 2 is the result of PCR. Lane 3 is gene ruler from Fermentaz. **B) **Plaque formation by recombinant λ phages on top agar.

Lambda ZAP^®^-CMV vector (Stratagene, USA) was used for construction of recombinant λ bacteriophages. The vector has potential characteristics for expression in eukaryotic cells. Eukaryotic expression of inserts is driven by the cytomegalovirus (CMV) immediate early (IE) promoter with the SV40 transcription terminator and polyadenylation signal (Figure 1).

### Bacterial strains

The RecA^- ^*E. coli *host strain XL1-Blue MRF' and VCS257 strain Δ(mcrA)183 Δ(mcrCB-hsdSMR-mrr)173 endA1 thi-1 recA1 gyrA96 relA1 lac [F'proAB lacIqZΔM15 Tn10 (Tet^r^)] Su^-^(nonsuppressing) λr is supplied with the Lambda ZAP-CMV XR predigested vector kit and Lambda ZAP-CMV XR Gigapack^® ^cloning kit for λ phage amplification and titration purposes. The *E. coli *DH5α were used as host cells during the cloning experiments and for propagation of the plasmids. Bacterial strains were routinely grown at 37°c in LB broth (Gibco BRL) or on agar containing medium, supplemented with 50 μg/ml ampicilin, whenever required.

### Preparation of plasmid DNA

The E7 gene with flanking EcoRI and Xho I sites, respectively, was synthesized by ShineGene (Shanghai ShineGene Molecular Biotech, Inc) and cloned into pUC18 vector.

*E. coli *bacterial strain DH5α was transformed with plasmid DNA encoding EGFP (pEGFP-C1) (BD Biosciences Clontech, GenBank Accession # U55763) (Figure 1C) and pUC18 incubated in selective Luria-Bertani (LB) medium and extracted from the culture pellets using a QIAGEN endotoxin free Mega Plasmid kit (QIAGEN, Hilden, Germany) according to the manufacturer's instructions. The purity and identity of the plasmid was confirmed by agarose gel electrophoresis.

### Construction of λZAP-CMV vectors

EGFP-Lambda ZAP-CMV DNA was constructed by subclonning of the EGFP fragment from pEGFP-C1 (employing primers eGFP-up: 5'-GTAGAATTC(*EcoRI*)ATGGTGAGCAAGGGCGAGG-3' and eGFP-down: 5'-GACCTCGAG(*XhoI*)TTACTTGTACAGCTCGTCC-3') into the corresponding sites in lambda ZAP-CMV DNA [26]. For constructing λ-Zap HPV-16 E7, The HPV-16 E7 gene was excised from the pUC18 vector using suitable restriction enzymes to be ligated into lambda ZAP-CMV DNA. For ligation, we used an equal molar ratio (or less to prevent multiple inserts) of the inserts according to the kit instruction. The pBR322 (as a positive control of ligation) HPV-16 E7 and EGFP inserts were ligated into Lambda ZAP-CMV vector at a volume up to 2.5 μl using 2 U of T4 DNA ligase (Fermentaz) and 0.5 μl of 10 mM ATP (pH 7.5). For packaging of lambda pages, the ligated DNA immediately was added to the packaging extract. Also, 1 μl of the control ligation was added to a separate tube of packaging extract. Then, the tube was stirred with a pipette tip to mix well. The tube was spined quickly for 3-5 seconds. The tube was incubated at room temperature (22°C) for 2 hours. Five hundred micro liter of phage buffer (i.e., SM buffer included 5.8 g NaCl, 2.0 g MgSO_4_·7H2O, 50.0 ml 1 M Tris-HCl (pH 7.5), 5.0 ml 2% (w/v) gelatin up to one liter distilled water) was added to the tube. Then, 20 μl of chloroform was added and mixed the contents of the tube, gently. The tube was spined briefly to sediment the debris. The supernatant containing the phage was ready for titteration. The supernatant can be stored at 4°C for up to 1 month.

### DNA-packaging efficiency by Gigapack

For measurement of the interactions between DNA and Gigapack, ethidium bromide (EtBr), a DNA-intercalating dye, was used to examine the association of DNA with the packaging extract [27,28]. A solution of 400 ng/ml EtBr in HBG (20 mM HEPES, 5% (V/V) glucose, pH 7.4) was prepared with further addition of 10 μg/ml of DNA-Packaging extract. The fluorescence intensity of EtBr was measured at an excitation wavelength 510 nm and emission wavelength 590 nm with a 10-nm slit using spectrofluorimeter RF-500 (Shimadzu, Japan) and fluorescence was set to 100%. All measurements were done in triplicate.

### Tittering assembled-phage nanobioparticles

Cultures of XL1-Blue MRF' and VCS257 cells were prepared in LB broth supplemented with 0.2% (w/v) maltose (Merck) and 10 mM MgSO_4 _and then incubated at 37°C for 4-6 hours (OD_600 _of 1.0) with vigorous agitation. The bacteria were pelleted. Each cell pellet was gently resuspended in 25 ml sterile 10 mM MgSO_4_. The bacterial cells were diluted to an OD_600 _of 0.5 with sterile 10 mM MgSO4. For titration, the following components were mixed: One microliter of a different dilution of the final packaged reaction and 200 μl of XL1-Blue MRF' cells at an OD_600 _of 0.5 were mixed. The cell concentration was calculated, assuming 1 OD_600 _= 8 × 10^8 ^cells/ml.

The phage and the bacteria were incubated at 37°C for 15 minutes with intermittent shaking to allow the phage to attach to the cells. NZY media (Sigma Ltd., UK) was added to top agar, melted and cooled to 48°C, and plated immediately onto dry, prewarmed NZY agar plates. The plates were allowed to set for 10 minutes. Then the plates were inverted and incubated at 37°C. Plaques would be visible after 6-8 hours. The plaques counted and determined the titer in plaque-forming units per milliliter (PFU/ml).

### Phage amplification

Bacteriophage stocks were generated by picking a well isolated plaque and placing the agar/agarose containing the zone of lysis in SM solution. Resulting stock solution were used to prepare liquid cultures of the bacteriophage. For amplification, culture of XL1-Blue MRF' cells was grown in LB broth with supplements, and then diluted the cells to an OD_600 _of 0.5 in 10 mM MgSO_4_. XL1-Blue MRF' cells were infected at an OD_600 _of 0.5 with bacteriophage in 50-100 μl of SM solution.

After 20 minutes absorption at 37°C, 4 ml of NZY medium was added, prewarmed to 37°C, and incubated the culture with vigorous agitation until lysis occurred (usually 8-12 hours at 37°C). Then chloroform was added to the lysates and continued incubation for 15 minutes at 37°C. The culture was centrifuged at 11000 × g for 10 minutes at 4°C. The supernatant was recovered, 50 μl of chloroform added, and then stored the phage stock.

### Purification of λ-phage nanobioparticles

Lysed cultures were cooled to room temperature. Deoxyribonuclease I and Ribonuclease A (both Sigma Ltd., UK) were added to a final concentration of 1 μg/ml and incubated for 30 min at room temperature. NaCl was added to 1 M, and flasks were left to stand on ice for an hour. Cell debris was removed by centrifugation at 11000 × g for 10 min at 4°C and the collected supernatants were pooled. Solid Polyethylene glycol (PEG, Sigma Ltd., UK) was added to a final concentration of 10% (w/v), dissolved slowly at room temperature, then the flasks incubated at 4°C for at least 1 h. The precipitated bacteriophage particles were recovered by centrifugation at 11000 × g for 10 m at 4°C and the pellet re-suspended in SM buffer. An equal volume of chloroform was added, and the culture was centrifuged at 300 × g for 15 min at 4°C. The upper aqueous phase was then removed and the bacteriophages were pelleted by centrifugation at 11000 × g for 2 h at 4°C.

The bacteriophage pellet was re-suspended in 1-2 ml of SM buffer at 4°C overnight, pelleted once more, and then re-suspended in SM prior to storage or further manipulation.

### Transduction of mammalian cell lines

Four cell types were selected in transduction experiments: COS-7 simian-derived kidney, CHO Chinese hamster ovary, TC-1 C57BL/6 mouse lung epithelial cells (Part of the Johns Hopkins Special Collection) and HEK-293 human embryonic kidney epithelial cell lines were cultured in RPMI 1640 (Gibco BRL, Paisley, UK) supplemented with 10% fetal bovine serum, and 100 U/ml penicillin, 100 μg/ml streptomycin (components from Sigma, Germany), and 2 mM L-glutamine. After overnight incubation, culture medium was removed and replaced with serum-free medium 30 min prior to the addition of λ-phage nanobioparticles containing enhanced green fluorescent protein (EGFP). Phage nanobioparticles at a multiplicity of infection (M.O.I) of 10^6 ^were added to mammalian cell lines. Transduction of target cells was enhanced using spinoculation or centrifugal enhancement after addition of phage particles [26]. Briefly, cell cultures were centrifuged at 1000 × g for 10 min at 37°C. Following centrifugation, cells were incubated for an additional 10 min at 37°C and phage containing media was removed and cell were washed twice with 1× PBS and then incubated in complete media. After 36 hours post infection, the cells were examined by fluorescent microscopy. Transfected COS-7 cells by wild type λ-phages were used as negative control.

### SDS-PAGE and Western blot

To confirm the expression of recombinant HPV E7 in the cell lines, western blot analysis was performed on the extracted total protein. CHO Cells growing on 6-cm plates were infected with lambda ZAP-E7 particles or wild λ-particles as a control at a multiplicity of infection (M.O.I) of 10^6 ^and allowed to express the protein for 48 h. Then, the cells were washed twice with ice cold phosphate-buffered saline (PBS) and lysed in sodium dodecyl sulfate (SDS) loading buffer containing 1 mM dithiothreitol. Cellular proteins were separated on 15% polyacrylamide gels by SDS-polyacrylamide gel electrophoresis (PAGE), blotted onto polyvinylidene difluoride membranes (Roche, Germany), and hybridized with the monoclonal HPV-16 E7 mouse antibody (Abcam, UK), followed by detection with goat anti-mouse secondary antibody conjugated to alkaline phosphatase (Sigma, St Louis, MO) in secondary antibody solution. Color was developed by incubating the membrane in alkaline phosphate buffer containing NBT (nitro blue tetrazolium) and BCIP (bromochloroindolyl phosphatase.

### *In vitro *internalization assay

Cells were seeded at 1 × 10^5 ^cells per well in a 96 well flat bottom plate and 1 × 10^11 ^plaque forming unite (PFU) (M.O.I multiplicity of infectious 1 × 10^5^) of EGFP-λ-phages were added. Plates were incubated at 37°C for 4 hours. Following incubation, the cells were trepsinized and resuspended in 1× PBS and transferred to a microcentrifuge tube. The cells were pelleted by centrifugation and resuspended in an ice cold wash (0.3 M acetic acid, 0.5 M NaCl, pH 2.5) three times to remove any phage bound to the cell exterior. After the acid wash, the cells were washed once in 1× PBS, pelleted and resuspended in 1× TE (Tris-EDTA) and then manually lysed by passing through a 22G insulin syringe 10 times to sheer the cells. The lysed cells were centrifuged to remove cellular debris and phage containing supernatant. Internalized phages in cell lysates were then quantified by titration on *E. coli *cells.

### Tumor Therapy Assay

C57BL/6 mice (6-8 weeks old) were purchased from the Pasteur Institute (Karaj, Iran). Mice were housed for 1 week before the experiment. All experiments were done according to the guidelines for the care and use and the guidelines of the laboratory animal ethical commission of Tarbiat Modares University.

TC-1, was derived from primary epithelial cells of C57BL/6 mice cotransformed with HPV16 E6 and E7 and activated c-Ha-ras oncogene. TC-1 cell line which is HPV-16 E7+ was used as a tumor model in an H-2b murine system. For in vivo therapeutic experiments, C57BL/6 mice (seven per group) were challenged by subcutaneous injection in the right flank with TC-1 cells 2 × 10^5 ^suspended in 100 μl PBS. After one week, the mice were immunized with 2 × 10^12 ^particles of recombinant λ-ZAP E7 phage, wild λ-ZAP phage (phage control) and PBS (negative control) via subcutaneous injection. Mice received two boosts with the same regimen 1 and 2 weeks later.

Subcutaneous tumor volume was estimated according to Carlsson's formula. Hence, the largest (a) and the smallest (b) superficial diameters of the tumor were measured twice a week and then the volume (V) of the tumor was calculated (V = a × b × 1/2). To compare results between the different groups, a one way ANOVA test was used. The statistical software SPSS 11.0 was utilized for statistical analyses. Differences were considered statistically significant when P value < 0.05. All values were expressed as means ± S.D.

## Results

### Confirmation of EGFP and E7 lambda cassettes

Presence of EGFP and E7 genes in packaged phages was confirmed by polymerase chain reaction using eGFP primers and E7 primers, respectively. Also, the plaque formation of phages on top agar approved subclonning and ligation steps (Figure 1).

### Packaging efficiency

**Figure 2:**
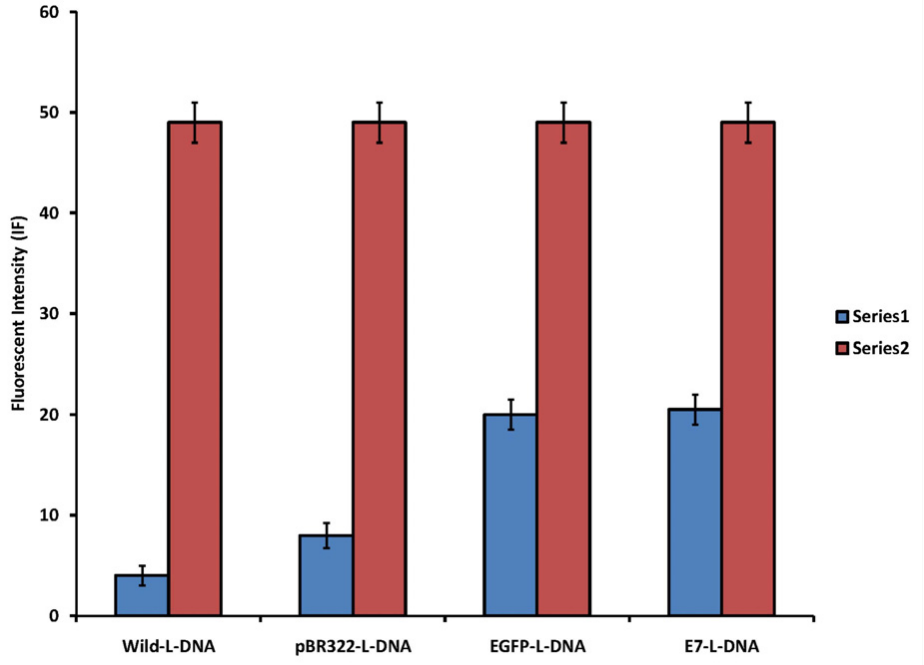
**Packaging efficiency of wild-λ-DNA, pBR322-λ-DNA, EGFP-λ-DNA, and E7-λ-DNA before (series 2) and after (series 1) packaging using Gigapack**.

Packaging analysis using EtBr-dye indicates an equal fluorescent intensity (IF) for lambda DNAs (wild-L-DNA, pBR322-L-DNA, E7-L-DNA and EGFP-L-DNA) before packaging by Gigapck contained lambda protein lysates. The DNAs showed different behaviors after EtBr intercalation (Figure 2). The packaging efficiencies for pBR322-L-DNA, EGFP-L-DNA, and E7-L-DNA were respectively measured as 91%, 64%, and 63.3% in comparison to wild-L-DNA (100% packaging efficiency).

### Gene delivery and expression

**Figure 3:**
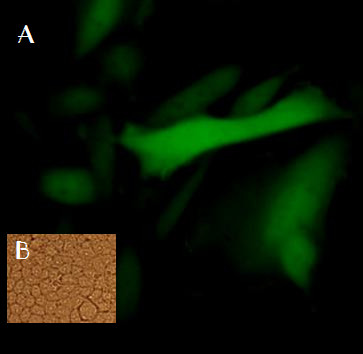
**A. CHO cells transfected by EGFP-λ-phage nanobioparticles using fluorescent microscopy**. Four cell types COS-7, CHO, TC-1 and HEK-293 were transfected by EGFP-λ-phages. The best GFP expression was observed after 48 hours by fluorescent microscopy 48 hours later in CHO cells. B. CHO experimental control.

To test functionality of the EGFP expression cassette in recombinant λ phages, four different cell lines were transduced by phage particles and then examined the cells by fluorescent microscopy after 36 h. The best GFP levels were detected in CHO cell line (Figure 3). Thus, the mammalian GFP expression cassette in λ phages was functional and λ-EGFP phage transduced an EGFP gene in the CHO cell line, efficiently. The lack of GFP signal from a functional expression cassette within the phage genome suggests that the phage may not be efficiently internalizing into the other cell lines

### SDS-PAGE and Western blot

**Figure 4:**
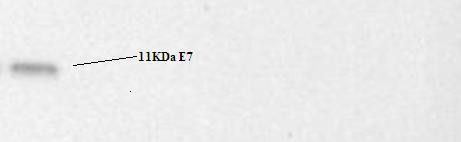
**SDS-PAGE and Western blot analyses of CHO cells infected with E7-λ-phages**. After overnight incubation, the cellular proteins were extracted and analyzed by SDS-PAGE and immunoblotting.

Western blot analysis was performed on lysates (10^6 ^cells), 48 hr post transfection, and it showed a signal corresponding to the E7 protein (11 kDa; Figure 4).

### Internalization assays

**Figure 5:**
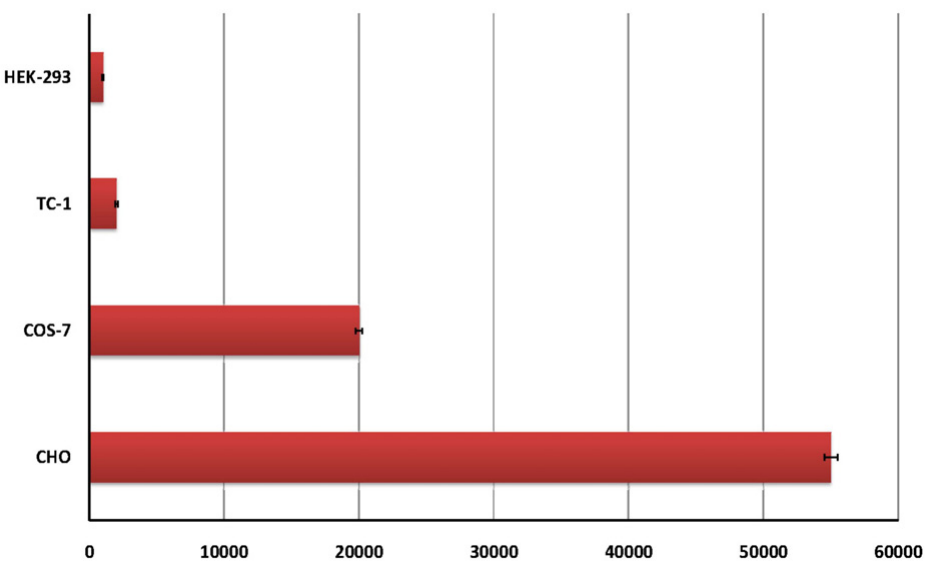
**The internalizing analyses for four cell lines (COS-7, TC-1, HEK-293, and CHO)**.

The internalizing analyses on four cell lines (COS-7, TC-1, HEK-293, and CHO) showed that the titer of internalized phages is different according to the kinds of cells. Meanwhile it was shown that wash buffer containing Tris-EDTA didn't have any side effects on phage titer (Data not shown). The internalizing titer was calculated 2 × 10^4 ^PFU for COS-7 cells and 5.5 × 10^4 ^PFU for CHO cells, whereas the range was 2 × 10^3 ^and 1 × 10^3 ^for TC-1 and HEK-293 cells, respectively. It seems that these variations in internalizations maybe because of the source of the cells (Figure 5). As a consequent, COS-7 cells with fibroblastic source had optimum capability for phage internalization and gene expression, whereas the two other cells with epithelial sources had fewer capabilities for phage entrances and gene expressions.

### Therapeutic antitumor assay

**Figure 6:**
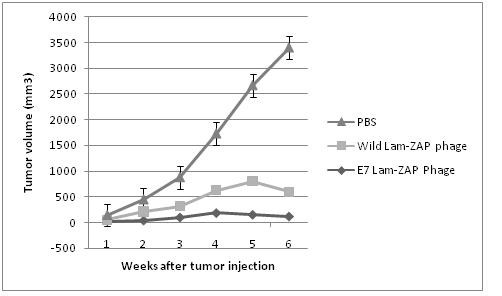
**Therapeutic vaccination against TC-1-induced tumors**. Mice were inoculated with 2 × 105 TC-1 tumor into the right flank and then treated with recombinant λ-ZAP E7 phage, Wild λ-ZAP phage (phage control) and PBS (negative control) 7 days after inoculation. Mice were monitored for evidence of tumor growth by palpation and inspection twice a week. For determining of tumor volume, each individual tumor size was measured. Line and scatter plot graphs depicting the tumor volume (mm3) are presented. The data presented is a representation of two independent experiments.

To determine anti-tumor activity of recombinant λ-ZAP E7 phage could, we performed an in vivo tumor treatment experiment using a previously characterized E7-expressing tumor model, TC-1 [19]. Tumors were measured twice a week once the tumors became palpable. Controls consisted of unimmunized mice challenged with tumors. The tumor volume was monitored up to 30 days after the tumor challenge. As shown in Figure 6, mice treated with recombinant λ-ZAP E7 phage significantly reduced tumor volume, compared to mice treated with the wild λ-ZAP phage and PBS (P < 0.05). These results indicate that vaccination with recombinant λ-ZAP E7 phage could induce significant therapeutic anti-tumor effects than vaccination with control groups.

## Discussion

Many cancer vaccines currently under investigation are based on recombinant carriers such as viruses and bacteria [29]. In animal models, these vaccines can elicit powerful immune responses that lead to tumor cell destruction, but a number of obstacles remain in the translation of these strategies to the clinic [30]. One of the major difficulties is high pre-existing, neutralizing titers to vaccines based on human viruses and bacteria, likely as the result of ubiquitous exposure with this agents [31]. One way of circumventing pre-existing immunity is the use of viruses whose natural hosts are non-mammalian such as bacteriophages.

In the present study, the construction and characterization of recombinant lambda bacteriophages for gene delivery and expression in mammalian cells were reported as a cancer-candidate vaccine. There are few reports about using lambda phages for this purpose, but some aspects of these biological tools have not been studied so far [32]. For example, like other nanocarriers, it is interesting to measure the packaging efficiency of the phage lysates when they package different sizes of the lambda DNAs. Therefore, the packaging efficiency of λ-phage nanobioparticles was estimated by different sizes of DNAs. Higher packaging efficiency was obtained from the lambda-based cassette consisting of pBR322 with a larger DNA in comparison to the EGFP DNA. It was demonstrated that the maximum packaging of lambda lysates for the vector contained the wild type of lambda-phage DNA (figure 2).

EGFP expression in CHO cells indicated the efficiency of lambda phages as biological nanocarriers in gene delivery to mammalian cells.

Results from the internalization assays (figure 5) showed the phage particles could be internalized more efficiently in the fibroblastic cells (i.e., COS-7 and CHO). However, the epithelial cells (such as TC-1 and HEK-293) had less capability for macropinocytosis of phage nanobioparticles. This means, although the significant numbers of cells internalize the phages, the cells do not essentially express the EGFP gene. The fact suggests the transduction frequency of phages is limited by one or more post-uptake events [21,33].

In vivo experiment demonstrated efficient therapeutic anti-tumor effects of recombinant lambda phage containing HPV-16 E7. The antitumor cell-mediated immune responses induced by recombinant phages are likely to play a role in this function. It seems that Natural immunostimulatory of the lambda phage enhance the anti-tumor effects of recombinant phage.

March et al described lambda-gt11 (having CMV promoter) vector as gene delivery system and vaccine [21], the lambda-based vector presented here for the first time uses lambda ZAP CMV vector. Further researches aimed at improving their efficiency, lysosomal escape, transgene nuclear localization, or transgene cassette stability during cell division will contribute to the production of more effective tools for gene transfer experiments, eventually providing efficient eukaryotic viral vectors. Our work has increased the understanding of lambda-phage-mediated gene transfer and suggests new approaches may lead to the design of potential phage nanomedicines for novel phage-based drugs and vaccines in future[21-23].

## Abbreviations

PFU: Plaque forming unit; M.O.I.: Multiplicity of infection; PBS: phosphate-buffer saline; RPMI 1640: Roswell Park Memorial Institute; MCS: Multiple cloning site.

## Competing interests

The authors declare that they have no competing interests.

## Authors' contributions

AG carried out the molecular cloning and characterization and drafted the manuscript. HS participated in the design of the study and drafted the manuscript. PG participated in the design of the nanobioparticles, drafted the manuscript and performed the statistical analysis. ZH conceived of the study, and participated in its design and coordination. SR carried out the tumor assays. FR conceived of the study and participated in its design and coordination. All authors read and approved the final manuscript.
